# Paraneoplastic Pemphigus Associated with Follicular Dendritic Cell Tumor in the Mediastinum

**DOI:** 10.1155/2016/6901539

**Published:** 2016-04-11

**Authors:** Aparna Mullangath Prakasan, Anne Jennifer Prabhu, Kanmani Velarasan, Selvamani Backianathan, Thomas Samuel Ram

**Affiliations:** ^1^Ida B Scudder Cancer Centre, Radiation Oncology Unit 1, Christian Medical College, Ida Scudder Road, Vellore, Tamil Nadu 632004, India; ^2^Department of Pathology, Christian Medical College, Ida Scudder Road, Vellore, Tamil Nadu 632004, India

## Abstract

Paraneoplastic Pemphigus (PNP) is an autoimmune bullous disease characterized by severe stomatitis, polymorphous skin eruptions, and underlying neoplasms. Diagnosis of cutaneous paraneoplastic disorders requires high index of suspicion. We describe a patient with PNP associated with follicular dendritic cell (FDC) tumor in the mediastinum, a rare neoplasm originating from follicular dendritic cells. Its management requires identification of underlying malignancy and treatment of the same. Our patient showed remission of PNP upon excision of the tumor and remained disease-free for 8 years.

## 1. Introduction

Follicular dendritic cell tumors are rare neoplasms (0.4% of soft tissue sarcomas) [1], also called follicular dendritic reticulum cell tumor or follicular dendritic cell sarcoma. The behavior of these tumors is more like that of a low-grade soft tissue sarcoma than a malignant lymphoma and is characterized by local recurrences in 36% and distant metastases in 28% of cases to liver or lung [2]. They are often misdiagnosed. Immunohistochemical markers CD21 and CD35 positivity is necessary for diagnosis [3]. Complete surgical resection is the primary modality of treatment. The role of adjuvant treatment is not clearly defined. Patients with risk features like high grade, large tumor size (>5 cm), recurrent tumor, and incomplete resection will benefit from adjuvant treatment.

So far only 4 cases of FDC arising from Castleman's disease with Paraneoplastic Pemphigus (PNP) have been reported in literature [4–7].

## 2. Case Report

A 39-year-old gentleman presented with persistent oral erosions and skin vesicles over the left forearm for 4 months in June 2007. There was associated voice change, foreign body sensation in eyes, and weight loss. Clinical examination revealed multiple erosions over lips, hard palate, and buccal mucosa with hemorrhagic crusts over the lips. There was a single resolving vesicle over left forearm. Nikolsky and bulla sign were negative. There was no palpable lymphadenopathy. The rest of the systemic examination was unremarkable.

Perilesional lip biopsy under Direct Immunofluorescence (IF) showed fishnet pattern fluorescence for IgG in the lower third of epidermis and coarse granular contiguous positivity for C3 in the basal layer of keratinocytes. It was negative for IgA and IgM. He was started on Prednisolone and Azathioprine for one month, but his lesions did not improve.

**Figure 1:**
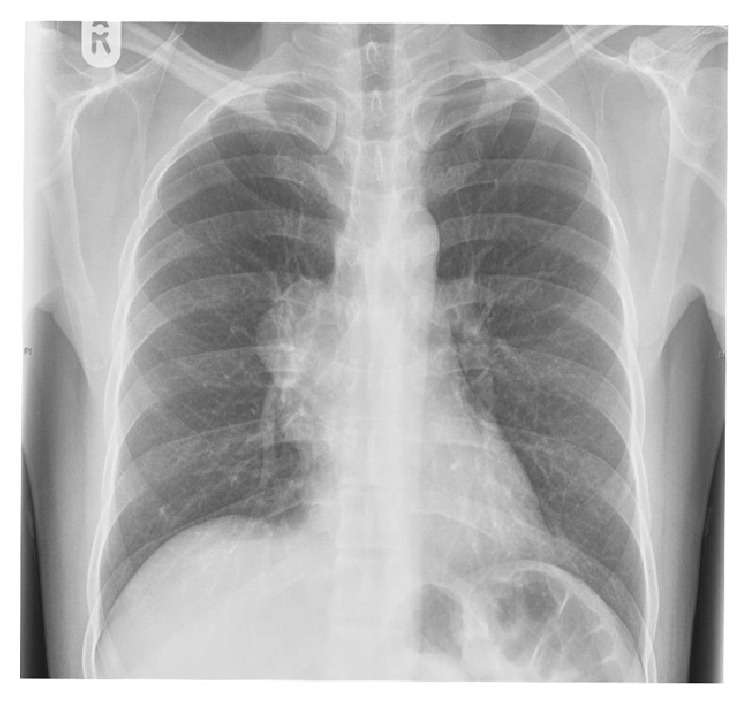
Chest X-ray showing right sided hilar mass.

**Figure 2:**
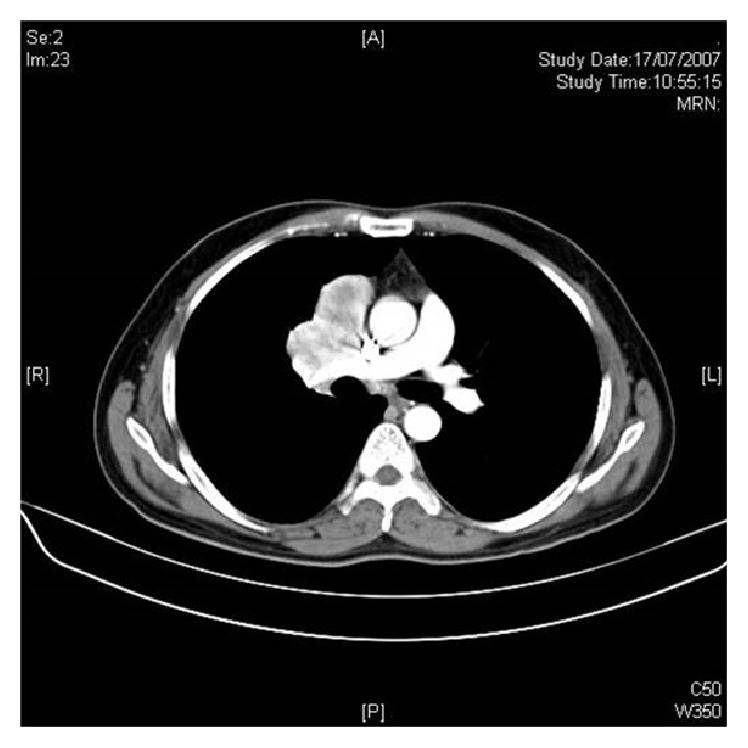
CT thorax showing densely enhancing anterior and middle mediastinal mass.

Routine chest X-ray done showed right sided hilar mass (Figure 1). Further evaluation with CT thorax revealed 6 × 3.8 cm, densely enhancing mass in the anterior and middle mediastinum (Figure 2).

**Figure 3:**
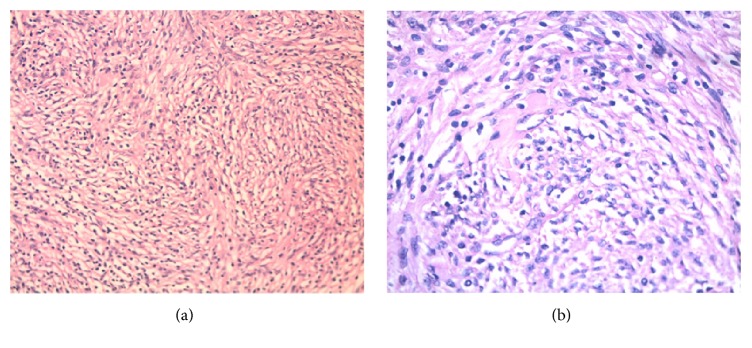
Circumscribed spindle cell tumor composed of interlacing fascicles with storiform pattern and sprinkling of lymphocytes on microscopy.

He had thoracotomy and debulking of the mass of 6 × 5 cm tumor in the right lobe of thymus, adherent to right phrenic nerve. There were no enlarged lymph nodes. Gross specimen showed an ill-defined tumor of size 2.5 × 3.5 × 3 cm with homogenous yellowish tan surface. Microscopy showed circumscribed spindle cell tumor composed of interlacing fascicles with storiform pattern in a background of lymphocytes (Figure 3). Immunohistochemistry revealed cells positive for CD21 and CD35 leading to a pathological diagnosis of follicular dendritic cell tumor.

Patient had 2 major criteria of PNP (a) polymorphic mucocutaneous eruption and (b) concurrent internal neoplasia and 2 minor criteria (a) Direct IF showing intercellular and basement membrane staining and (b) Indirect IF staining with rat bladder epithelium with intracellular IgG staining positive and diagnosed to have FDC tumor of the anterior mediastinum with Paraneoplastic Pemphigus.

**Figure 4:**
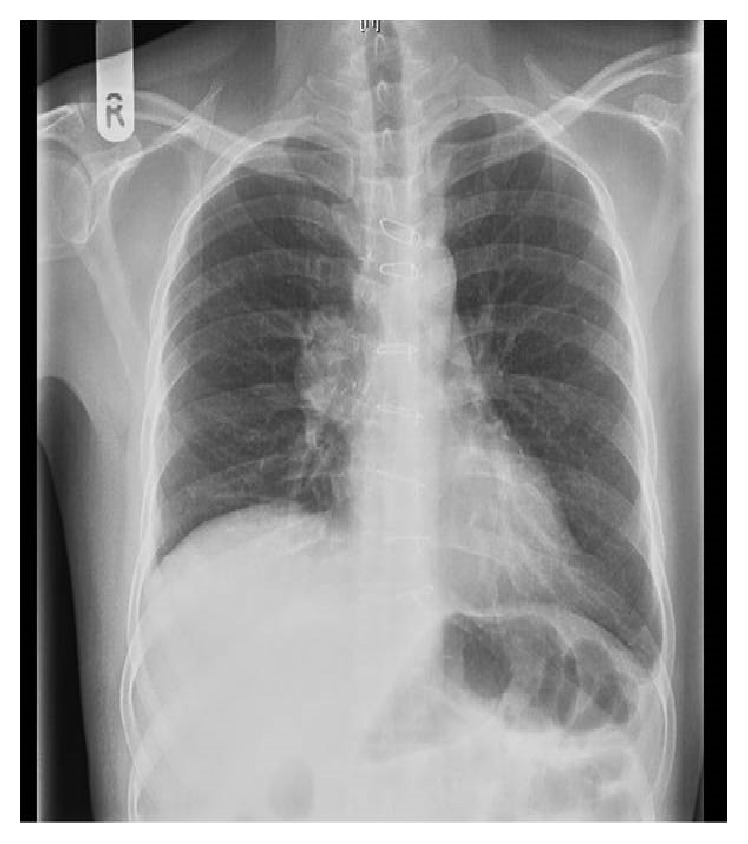
Postoperative chest X-ray showing residual mass in the anterior mediastinum.

**Figure 5:**
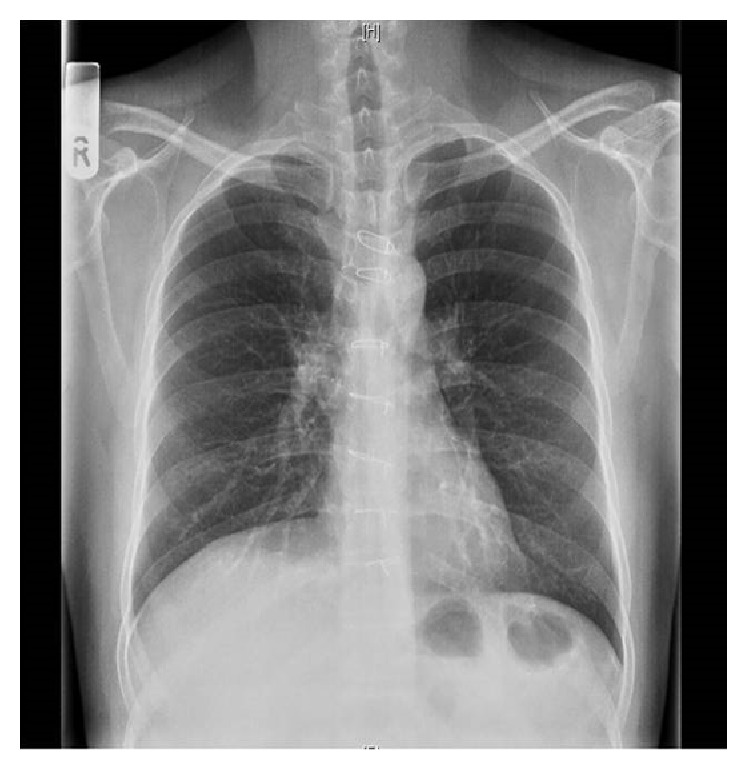
No residual lesion on chest X-ray on follow-up.

He received postoperative radiation therapy to the residual mass in the anterior mediastinum (Figure 4). He was treated with conventional anteroposterior-posteroanterior (AP-PA) technique, using 6 MV X-rays, 45 Gy in 25 fractions to midplane, and 5 fractions per week over 5 weeks. The skin and oral lesions subsided at the end of treatment. At 8-year follow-up, he is free of disease (Figure 5).

## 3. Discussion

Follicular dendritic reticulum cells are found in B cell zones, particularly in germinal centers. They are nonlymphoid, nonphagocytic cells, and main function of this cell is to capture and present antigens and immune complexes. Most commonly affected sites are lymph nodes and extranodal sites including liver, oral cavity, bowel, and spleen [8]. Intra-abdominal location, size >5 cm, 6 or more mitotic figures/10 HPF, atypia, and coagulative necrosis are associated with poor prognosis [9]. Differential diagnosis includes other histiocytic and dendritic cell neoplasms, fibroblastic reticulum cell sarcoma (expresses desmin, SMA, and cytokeratins), inflammatory pseudotumor (expression of FDC cell markers is weak), myofibroblastic tumors (positive for muscle specific actin), lymphoepithelioma-like carcinoma, malignant melanoma, thymoma, meningioma, malignant fibrous histiocytoma, and gastrointestinal stromal tumor.

WHO classifies dendritic neoplasm into Langerhans' cell histiocytosis, Langerhans cell sarcoma, interdigitating dendritic cell sarcoma, follicular dendritic cell sarcoma, and dendritic cell sarcoma, not otherwise specified.

According to Fonseca et al. [10] surgery is the primary modality of treatment for FDC. Benefit of consolidative radiotherapy in preventing local recurrence in patients with localized disease either completely or incompletely resected has been documented. Being so similar to lymphomas, physicians began using a common leukemia and non-Hodgkin's lymphoma chemotherapy regimen like CHOP and it had shown nondurable antitumor activity in FDC [10, 11].

Criteria for diagnosis of PNP include three major criteria or two major and two minor criteria. Major signs of Paraneoplastic Pemphigus include (1) polymorphic mucocutaneous eruption, (2) concurrent internal neoplasia, and (3) serum antibodies with a specific immunoprecipitation pattern. Minor signs of Paraneoplastic Pemphigus include (1) histologic evidence of acantholysis, (2) Direct IF showing intercellular and basement membrane staining, and (3) Indirect IF staining with rat bladder epithelium. Most constant laboratory finding is the characteristic immunoprecipitation pattern.

A review of 163 case reports of PNP by Kaplan et al. [12] reported that 84% of cases were associated with hematologic malignancies like non-Hodgkin's lymphoma [39%], Chronic Lymphocytic Leukemia [18%], Castleman's disease [18%], thymoma [6%], Waldenstrom macroglobulinemia [1%], Hodgkin's lymphoma [1%], and monoclonal gammopathy [1%]. The remaining 16% were associated with nonhematologic neoplasms such as epithelial origin carcinoma [9%], mesenchymal origin sarcoma [6%], and melanoma [1%]. Oral ulcerations were the presenting feature in 45% of all cases and appear to be a key feature of PNP.

Treatment of PNP includes identification of the underlying neoplasm and its treatment. In Leger and Picard review article, the overall survival for series of patients with PNP for 1 year, 3 years, and 5 years was found to be 49%, 41%, and 38%, respectively [13].

Differential diagnosis of anterior mediastinal mass with PNP includes lymphoproliferative disorders like thymic carcinoid, Castleman's disease, and nonlymphoproliferative disorders like sarcoma and lung carcinoma. Diagnosis of cutaneous paraneoplastic disorders requires high index of suspicion.

Our patient presented with PNP, an autoimmune bullous disease characterized by severe stomatitis and polymorphous skin eruptions occurring in association with various forms of underlying neoplasia. It is characterized by the presence of autoantibodies that react with intermediate filament-associated proteins in desmosomes and hemidesmosomes, desmoplakin, bullous pemphigoid antigen 1, and envoplakin.

In case reports with similar presentation (FDC with PNP), first patient's condition improved after excision of tumor [4] and second patient showed no improvement, possibly because of the occurrence of severe pulmonary involvement [5], third patient expired with septicemia following excision of the tumor [6], and fourth patient expired after 1st course of R-CHOP chemotherapy due to pseudomonas infection [7]. Our patient is found to be disease-free at 8-year follow-up.

Diagnosis of cutaneous paraneoplastic disorders requires high index of suspicion. Incorrect treatment can lead to adverse toxic effects of drugs. Its management requires identification of underlying malignancy and a multidisciplinary treatment of the same.
